# Time-Domain Filtering of Metasurfaces

**DOI:** 10.1038/srep16737

**Published:** 2015-11-13

**Authors:** Hiroki Wakatsuchi

**Affiliations:** 1 Center for Innovative Young Researchers, Nagoya Institute of Technology, Gokiso-cho, Showa, Nagoya, Aichi, 466-8555, Japan; 2 Department of Electrical and Electronic Engineering, Nagoya Institute of Technology, Gokiso-cho, Showa, Nagoya, Aichi, 466-8555, Japan

## Abstract

In general electromagnetic response of each material to a continuous wave does not vary in time domain if the frequency component remains the same. Recently, it turned out that integrating several circuit elements including schottky diodes with periodically metallised surfaces, or the so-called metasurfaces, leads to selectively absorbing specific types of waveforms or pulse widths even at the same frequency. These waveform-selective metasurfaces effectively showed different absorbing performances for different widths of pulsed sine waves by gradually varying their electromagnetic responses in time domain. Here we study time-filtering effects of such circuit-based metasurfaces illuminated by continuous sine waves. Moreover, we introduce extra circuit elements to these structures to enhance the time-domain control capability. These time-varying properties are expected to give us another degree of freedom to control electromagnetic waves and thus contribute to developing new kinds of electromagnetic applications and technologies, e.g. time-windowing wireless communications and waveform conversion.

Conventional materials available in nature vary their electromagnetic responses to incoming waves in accordance with the behaviours of composite molecules, which are angstrom scales and thus difficult to control in an artificial manner. On the other hand, periodically engineered structures, or the so-called metamaterials or metasurfaces^1^,^2^,^3^, enabled us to control their responses by arbitrarily designing sub-wavelength periodic units, which resonate in response to incoming waves. The use of artificial materials therefore gave us an additional degree of freedom to control electromagnetic properties independent of composite molecules, resulting in achieving even unusual properties including a negative or zero refractive index^2^ as well as a high impedance surface with an extremely thin dimension^3^. These exotic properties led to development of various new applications such as diffraction-limitless lenses^4^,^5^, cloaking^6^,^7^,^8^, extraordinarily thin absorbers^9^,^10^,^11^,^12^, antenna miniaturisation^13^,^14^, etc. The concept of artificial materials was not limited to electromagnetics but was also applied to other fields including acoustics^15^,^16^,^17^,^18^, thermodynamics^19^ and vibration engineering^20^,^21^. Such a capability of metamaterials and metasurfaces is known to be further extended by introducing nonlinear media or nonlinear circuits as they gave us a flexible tunability to vary electromagnetic properties^22^,^23^,^24^,^25^. Especially, recently developed metasurfaces that consisted of several circuit elements including schottky diodes exhibited new electromagnetic characteristics named waveform selectivity^26^,^27^,^28^,^29^. These waveform-selective metasurfaces allowed us to distinguish different waves even at the same frequency in response to their waveforms or pulse widths. As a consequence, these metasurfaces gave us another degree of freedom to control electromagnetic properties at the same frequency based on the new concept, namely, pulse width^28^,^30^. In the past studies these metasurfaces were demonstrated to effectively vary their *entire* absorbing performances for different pulses depending on how long the waves continued, although in fact these absorptances were time-varying and no longer remained the same in time domain. Therefore, such a new feature is expected to be exploited for filtering continuous sine waves even in time domain (Fig. 1). For this reason this study numerically clarifies the transient responses of waveform-selective metasurfaces as time-domain filters. Additionally, we present an idea to further extend the degree of the time-domain control by introducing extra circuit elements. These metasurfaces are expected to give us a new capability to control electromagnetic properties even in time domain, and thus contribute to developing new kinds of time-varying microwave applications and technologies, e.g. in wireless communications where incoming signals are selectively received just during a specific time period. In addition, these metasurfaces can be potentially exploited for converting a simple sine wave to a different waveform. Although the following study provides numerical simulation results only, similar effects are expected to be obtained experimentally as demonstrated earlier^26^,^27^,^28^,^29^. In realistic measurements, however, there might appear some minor difference in the operating frequencies of metasurfaces due to the presence of solder and parasitic components in diodes, which makes it slightly difficult to analyse the time filtering effects demonstrated below. Therefore, this study focuses only on numerical simulations for the sake of simplicity. Note that there exist several time-domain electromagnetic studies reported recently^31^,^32^,^33^,^34^. Compared to these studies, however, our study gives a unique way to control time domain response even at the same frequency with the same power level without any biasing source, which can be straightforwardly modified by the circuit parameters used.

To evaluate transient responses of our metasurfaces, this study uses a co-simulation method integrating a commercial electromagnetic (EM) simulator HFSS (version 2014) with a circuit simulator Designer (version 2014)^26^,^28^. In this method waveform-selective metasurfaces are firstly modelled and simulated in the EM simulator. A wave port is deployed to generate an incoming wave and observe an reflected wave, while lumped ports are used instead of all the circuit elements between conducting patches (PEC or perfect electric conductor). These lumped ports are then connected to the actual components terminated by ground in the circuit simulator where all the simulation results are obtained using the scattering information calculated in the EM solver beforehand. This simulation is thus effectively equivalent with directly connecting the circuit components to the metasurfaces in the EM solver. However, this method contributes to significantly reducing the calculation time and readily sweeping frequency and circuit parameters to optimise the structures at the expense of visualising spatial field distributions^35^.

Although more details can be seen in past studies^26^,^28^,^29^, the fundamental absorbing mechanism is understood in the following manner. As illustrated in Fig. 2, the waveform-selective metasurfaces studied here consist of periodically metallised patches (17 mm × 17 mm with small crops), ground plane and dielectric substrate (Rogers 3003). In each gap between metallised patches a set of four schottky diodes is deployed to play a role of a diode bridge, which rectifies incoming signals to an infinite set of frequency components. However, most energy is at zero frequency. In past studies this conversion to zero frequency was exploited together with the time-domain responses of capacitors and inductors to realise waveform selectivity. Specifically, capacitors temporarily stored the rectified energy and were gradually charged up due to the zero frequency component, while inductors prevented the incoming wave due to the presence of electromotive force that started disappearing if the wave continued long enough. For these reasons, if a capacitor was connected to a resistor in parallel inside a diode bridge (see the right of Fig. 2), the metasurface more effectively absorbed short pulses and transmitted long pulses even at the same frequency, while an inductor paired with a resistor in series more strongly absorbed long pulses than short pulses. In this study, however, these absorbing mechanisms are more straightforwardly exploited as time-domain filters by illuminating *continuous* sine waves on metasurfaces. As a result, a capacitor-based metasurface is expected to absorb an incoming wave more effectively *during* an initial time period and gradually reduce the performance. In contrast, an inductor-based metasurface shows a limited level of absorptance at first but more effectively absorbs *after* an initial time period.

While the metasurface simulated here is composed of a realistic substrate (Rogers 3003) and schottky diodes (Avago HSMS-2863/2864), only capacitors and inductors are modelled as ideal circuit chips for the sake of simplicity so that their package resonances are not taken into account. The capacitance and inductance are swept to investigate the relationship between these values and time-filtering effects, although the resistance value used is fixed at either 10 kΩ or 10 Ω depending on whether the resistors are paired with capacitors or inductors. The oscillation frequency of incoming waves *f*_0_ is set to 3.9 GHz with a power level of 0 dBm.

## Results

**Figure 3:**
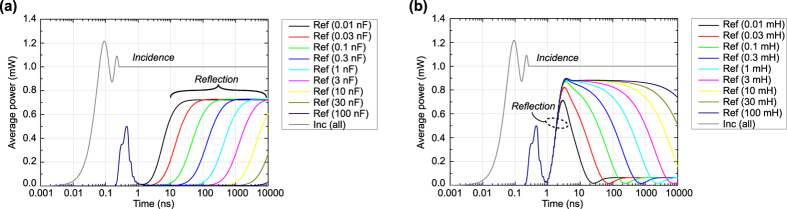
Transient incident and reflected powers averaged during last one cycle at each time. (**a**) Capacitor-based metasurface and (**b**) inductor-based metasurface with various time constants (specifically various capacitances and inductances). The resistance *R* for the capacitor-based metasurface was fixed at 10 kΩ, while that for the inductor-based metasurface was at 10 Ω. Note that the time constants *τ* for these metasurfaces are determined by *RC* and *L*/*R* where *C* and *L* are respectively capacitance and inductance. The oscillation frequency *f*_0_ was set to 3.9 GHz, which means that cycle *T* was approximately 0.26 ns.

Under these circumstances the transient responses of capacitor-based and inductor-based metasurfaces were calculated and plotted in Fig. 3. In this figure the incident and reflected powers *P*_*inc*_ and *P*_*ref*_ were obtained by averaging their instantaneous powers *P*_*inc*_*ins*_ and *P*_*ref*_*ins*_ over the last one cycle, namely, 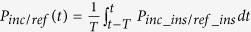, where *t* and *T* respectively denote time and cycle (i.e. *T* = 1/*f* where *f* is frequency and here 3.9 GHz). First of all, this figure shows that the average incident power curve reached the incoming power level set (i.e. 0 dBm) in one cycle from the beginning (*t* = *T* ~ 0.26 ns) and remained the same level, which indicates that the averaging was properly carried out. On the other hand, the reflected powers started increasing at *t* ~ 0.2 ns due to the distance between the metasurfaces and wave port where incident and reflected waves were observed. Between 0.2 and 3 ns all the reflected powers increased to approximately 0.5 mW and decreased to nearly 0.0 mW once. These were independent of the time constants used and were assumed to be due to the discontinuity of the incoming waveform, which generated not only the oscillation frequency *f*_0_ (3.9 GHz) but also a wide range of other spectrum.

**Figure 4:**
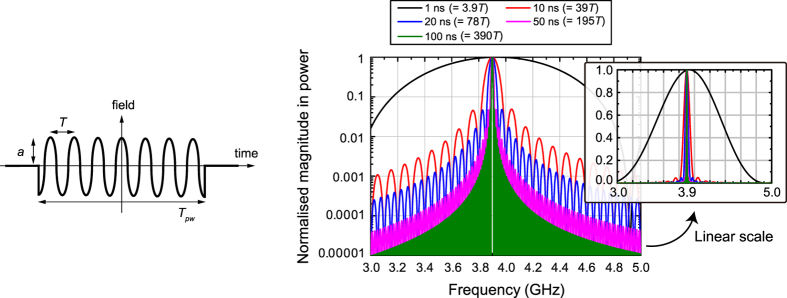
Spectrum of cosine pulse with various widths. To investigate the initial responses of the metasurfaces studied in Figure 3, the spectrum of a cosine pulse (oscillation frequency *f*_0_: 3.9 GHz) was analytically calculated by varying the pulse width *T*_*pw*_. If the pulse is long enough, the differences in the waveforms (i.e. cosine wave instead of sine wave) and time offset are expected to be negligible. The 1 ns pulse (only 3.9 cycles) contains a wide range of frequency components other than the oscillation frequency *f*_0_.

This influence is effectively evaluated in Fig. 4 where frequency spectrum for different widths of cosine wave pulses was analytically calculated. These curves were obtained by simply fourier-transforming a continuous cosine wave multiplied by a rectangular pulse that continued from *t* = −*T*_*pw*_/2 to *T*_*pw*_/2 where *T*_*pw*_ is the pulse width, namely,


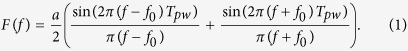


In this equation *a* represents the amplitude of the multiplied waveform. *F*(*f*) was then squared and normalised to yield the curves of Fig. 4. Although the sine wave was replaced with a cosine function and the oscillation period was offset by −*T*_*pw*_/2, the resultant fourier transform shows effectively the same result as the one using a sine wave that starts at *t* = 0 if the pulse width is long enough.

According to this figure, when the pulse width was set to 1 ns (which corresponds to only 3.9 *T* as *f*_0_ = 3.9 GHz), the resultant frequency spectrum exhibited a wide range of frequency components, which means that the 1-ns pulse contains a non-negligible level of other frequency components. Such an influence was assumed to appear in Fig. 3 as unusual reflection spikes (see between 0.2 and 1 ns). This influence, however, can be suppressed by increasing the pulse width. For example, at *T*_*pw*_ = 10 ns (i.e. at 39 *T*) the main spectrum curve remained at 3.9 GHz ± 0.1 GHz (i.e. *f*_0_ ± 2.6%). Moreover, doubling the pulse width (i.e. *T*_*pw*_ = 20 ns and 78 *T*) led to reducing the magnitudes of the frequency components lower than 3.8 GHz and higher than 4.0 GHz to 2% or less. Under this circumstance the bandwidth of the incoming pulse is assumed to be narrow enough compared to that of our metasurface used. For this reason, in Fig. 3 our metasurfaces exhibited a straightforward increase or decrease in the reflected powers after 3 ns. Additionally, Fig. 4 indicates that the time-filtering effects demonstrated below are obtained by sensing not the difference in the frequency spectrum but that in the duration time of the incident wave, as we use longer time scales.

**Figure 5:**
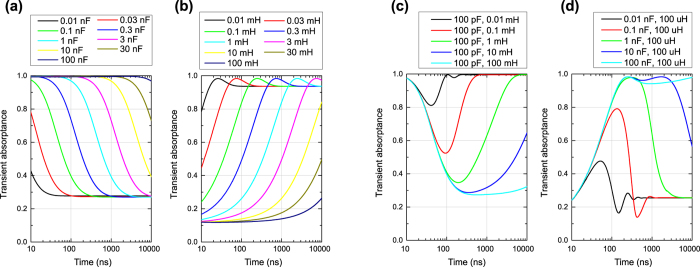
Transient absorbing performance of four fundamental metasurfaces. (**a**) Capacitor-based metasurface and (**b**) inductor-based metasurface. These metasurfaces respectively used fixed resistance values of 10 kΩ and 10 Ω. The capacitor- and inductor-based circuits were then combined in either parallel or series as (**c**) a parallel-type metasurface or (**d**) series-type metasurface.

The transient absorbing performances of the capacitor- and inductor-based metasurfaces of Fig. 3 are plotted in Fig. 5(a,b), respectively, where transient absorptance *A* was calculated from *A* = 1 − *P*_*ref*_/*P*_*inc*_. According to these figures the capacitor- and inductor-based metasurfaces dynamically varied the absorbing performances depending on the time constants *τ* (specifically *τ* = *RC* for the capacitor-based metasurface and *τ* = *L*/*R* for the inductor-based metasurface where *R*, *C* and *L* are respectively resistance, capacitance and inductance). For instance, with capacitors set to 1 nF the capacitor-based metasurface showed a strong level of absorptance until 50 ns. After this initial time period, however, the absorptance reduced to a lower level and reached approximately 30%. This time scale became 10 times larger/smaller by increasing/decreasing *τ* by a factor of 10. This indicates that the time scale is proportional to the time constant or more specifically capacitance *C* here. Similarly, the transient absorptance curve of the inductor-based metasurface was shifted by varying the time constant (again note that the time scale is proportional to *L*), although this structure gradually increased the absorbing performance. Another difference here is that each curve showed underdamping (e.g. around 2.5 *μ*s for *L* = 1 mH). The difference between overdamping and underdamping is assumed to be due to their damping coefficients, which are determined by the values of the circuit elements used. These two types of metasurfaces allow us to filter incoming signals in time domain as opposed to conventional filters, which sense the difference in frequency spectrum and thus do not exhibit such time-varying responses.

Next, combining these two circuit configurations either in parallel or in series leads to creating a time window during which the absorption level is temporarily reduced or enhanced as plotted in Fig. 5(c,d). This is because the parallel-type metasurface absorbs the incident wave during an initial time period due to the presence of the capacitor-based circuit, while later the absorbing mechanism of the inductor-based circuit activates so that this metasurface does not strongly absorb during a middle time period. For example, the parallel-type metasurface using 100 pF and 1 mH has nearly perfect absorption at both the beginning and end of the time period shown in Fig. 5(c). However, the metasurface permits reflection just during the limited period between 20 ns and 5 *μ*s as a time-domain filter. On the other hand, the series-type metasurface shows a limited level of absorptance at the beginning of the wave illumination due to the electromotive force of the inductors, while later the capacitors used are fully charged up and reduce the absorbing performance. As a consequence, this metasurface exhibits strong absorption during a limited time window during which both circuits effectively absorb the incoming wave. Note that these time windows can be readily controlled by selecting proper time constants for individual curves of capacitor- and inductor-based circuits.

Another point to note here is that these two metasurfaces also showed underdamping as seen in the inductor-based metasurface. Regarding these two cases the underdamping tended to appear if the absorptance curves did not reach the lowest or highest value of the original individual curves. For instance, the series-type metasurface using 10 nF and 100 *μ*H showed a strong level of absorbing performance between 300 and 2,000 ns in Fig. 5(d). When the capacitance was reduced to 0.1 ns, however, the absorptance started decreasing before reaching this level and then showed underdamping around 300 *μ*s.

This time-domain filtering capability can be more extended by introducing additional capacitor- and inductor-based circuits. Here it is important to consider the role of combining extra components either in *parallel* or in *series*. The former case, as seen in Fig. 5(c), creates another current path to potentially absorb the energy of incident wave and thus takes a stronger level of absorptance between the two individual curves, which is assumed to be like the “OR” operation of Boolean algebra. On the other hand, the latter case, as seen in Fig. 5(d), adds another circuit components on the same path to prevent the energy dissipation and thus takes a weaker level of absorptance between the two individual curves, which is assumed to be like the “AND” operation of Boolean algebra.

**Figure 6:**
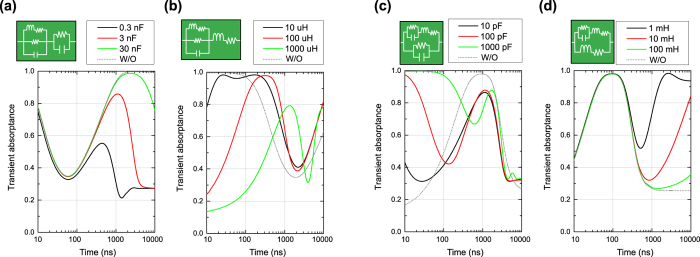
Transient absorptance of parallel- and series-type metasurfaces using either another capacitor-based circuit or inductor-based circuit. The parallel-type metasurface contained (**a**) an additional capacitor-based circuit or (**b**) inductor-based circuit in series, while the series-type metasurface contained (**c**) an additional capacitor-based circuit or (**d**) inductor-based circuit in parallel.

Taking account of these points, the absorptance of a parallel-type metasurface can be in part reduced by introducing additional circuit elements in *series* as plotted in Fig. 6(a,b). In these panels the right and left sides of the absorptance of the original parallel-type metasurface are reduced by using extra capacitors and inductors, respectively, which have lower absorptances than that of the original parallel-type metasurface. In contrast, the absorptance of a series-type metasurface can be enhanced by using an additional circuit configuration in *parallel* if it has a larger absorptance than that of the original series-type metasurface (Fig. 6(c,d)).

**Figure 7:**
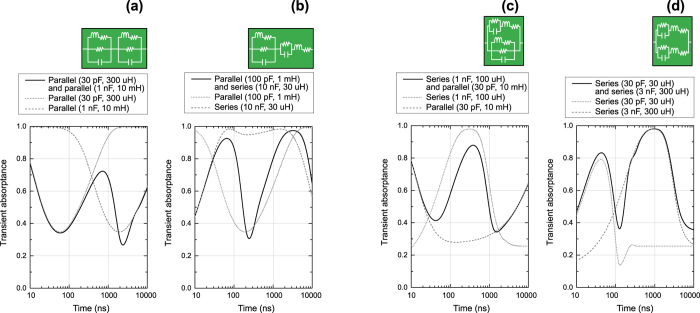
Transient absorptance of parallel- and series-type metasurfaces using either another parallel circuit or series circuit. The parallel-type metasurface contained (**a**) an additional parallel circuit or (**b**) series circuit in series, while the series-type metasurface contained (**c**) an additional parallel circuit or (**d**) series circuit in parallel.

More complex time-domain control is realised by using more than one of parallel or series circuit configurations. For example, Fig. 7(a,b) used a metasurface that connected a parallel circuit to either another parallel circuit or series circuit in series. In the former case, the use of two different parallel circuits that had different time windows to temporarily reduce absorptance during different time periods (i.e. the dotted and dashed curves in Fig. 7(a)) exhibited two absorptance dips in time domain (the solid curve in Fig. 7(a)). In the latter case, combining a parallel circuit with a series circuit led to creating a temporal reduction in the middle of the absorptance peak of the series circuit so that there appeared two transient absorptance peaks. Similar effects can be obtained by integrating a series circuit with either a parallel circuit or another series circuit in parallel. In Fig. 7(c) a parallel circuit showed an absorptance dip between 100 and 1000 ns (the dashed curve), which was enhanced again by using a series circuit (the dotted curve) that had an absorptance peak around 300 ns. As a consequence, this metasurface effectively showed two absorptance dips (the solid curve) similar with the ones seen in Fig. 7(a), although these absorbing mechanisms are different from each other. Moreover, Fig. 7(d) demonstrates another way to create two transient absorptance peaks (the solid curve) by combining two of independent series circuits in parallel (the dotted and dashed curves) (cf. Fig. 7(b)).

## Discussion

The time-filtering effects demonstrated in this study are limited to two transient absorptance peaks/dips or less. However, introducing more circuit components enables us to control the transient response in a more complex manner, resulting in a larger number of absorptance peaks or dips. From the realistic experimental viewpoint, however, under these circumstances there might not be an enough space to solder circuit components in each gap between conducting patches. In this case use of conducting vias might be helpful to connect these components underneath the ground plane. Additionally, in our numerical study we did not take account of the resonance of circuit chips for the sake of simplicity (i.e. capacitance and inductance were simply swept). In reality this factor may play an important role in the measurement, although the dominant frequency component of the current flowing into capacitors and inductors is at zero frequency due to the rectification process of diodes. Thus, this indicates that the influence of the frequency dispersion or package resonance of capacitor and inductor chips is relatively limited. A more important issue here lies in diodes as they are not capable of fully responding to high frequency signals compared to microwave signals, although some commercial diodes work even at W band. Moreover, in our study the input power was set to be large enough to turn on diodes, since otherwise they would not be able to rectify incoming signals and realise time filtering effects. Thus the relationship between the time filtering effects and input power level depends on what kind of diodes is used. Finally, the transient response of each metasurface demonstrated here is determined by the time constant(s) used, while the geometry of the metasurface determines the frequency response first.

## Conclusion

In summary we have numerically studied transient absorbing performances of recently developed waveform-selective metasurfaces in time domain. We have also introduced an idea to extend the time-filtering capability so that multiple transient absorptance peaks or dips can be readily designed. Conventionally, most electromagnetic materials exhibit different behaviours in response to different frequencies, although their responses generally remain the same in time domain. In contrast, our metasurfaces are capable of varying their responses even in time domain depending on how long the wave continues. Hence, these metasurfaces are expected to create new kinds of microwave applications and technologies that have an additional degree of freedom to control electromagnetic properties in time domain. For example, waveform-selective metasurfaces were already demonstrated to be able to vary a bit error rate in accordance with the pulse width of an incoming wave even at the same frequency^28^. Besides, an inductor-based metasurface has a limited level of absorption during an initial time period. Thus, within this limited time period, wireless communications may be permitted through short-pulse signals such as ones used for radar, ultra wideband communications and so on.

## Additional Information

**How to cite this article**: Wakatsuchi, H. Time-Domain Filtering of Metasurfaces. *Sci. Rep.* **5**, 16737; doi: 10.1038/srep16737 (2015).

## Figures and Tables

**Figure 1:**
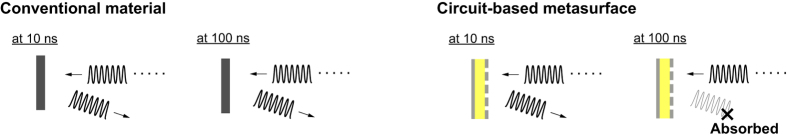
Image of time-varying response to continuous wave. Circuit-based metasurfaces studied here allow us to control their electromagnetic responses in accordance with the duration time of the incident wave, which is determined by their time constants. As a consequence, these structures behave as time-domain filters unlike conventional circuit filters, which behave differently in response to the difference in frequency spectrum.

**Figure 2:**
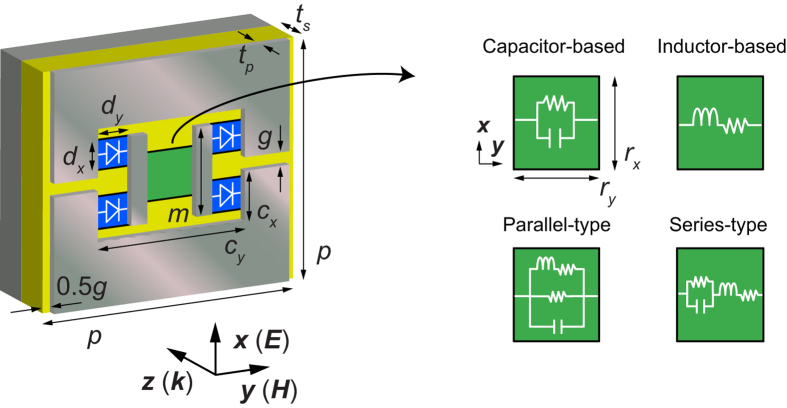
Periodic unit of metasurface model. Periodic boundaries were applied for *x*- and *y*-axis directions to model an infinite array of the periodic structure. The metasurface was composed of conducting patches (grey), substrate (Rogers 3003) (yellow), ground plane (grey) and several circuit elements. A set of four schottky diodes (Avago HSMS-2863/2864) (blue) was deployed in each gap between patches to play a role of a diode bridge. Note that current flows rightwards in the green area, where other circuit components were used to determine the type and response of the time-filtering function. Each dimension was given from *c*_*x*_ = 1.7, *c*_*y*_ = 7.6, *d*_*x*_ = 0.5, *d*_*y*_ = 1.3, *g* = 1.0, *l* = 2.4, *p* = 18.0, *r*_*x*_ = 2.0, *r*_*y*_ = 1.0, *t*_*p*_ = 0.017 and *t*_*s*_ = 1.524 (all in mm).
